# Determinants of Nurses’ Continuance Intention to Use Mobile Health Apps in Clinical Nursing Practice: Structural Equation Modeling to Extend the Expectation-Confirmation Model

**DOI:** 10.2196/68048

**Published:** 2025-10-31

**Authors:** Narjes Mirabootalebi, Felix Holl, Zahra Meidani, Fatemeh Rangraz Jeddi, Zahra Tagharrobi, Hossein Akbari, Walter Swoboda

**Affiliations:** 1 Health Information Management Research Center, Kashan University of Medical Sciences, Kashan, Iran; 2 DigiHealth Institute, Neu-Ulm University of Applied Sciences, Neu-Ulm, Germany; 3 Leibniz ScienceCampus Digital Public Health, Bremen, Germany; 4 Department of Health Information Management and Technology, Allied Medical Sciences Faculty, Kashan University of Medical Sciences, Kashan, Iran; 5 Trauma Nursing Research Center, Kashan University of Medical Sciences, Kashan, Iran; 6 Social Determinants of Health (SDH) Research Center, Kashan University of Medical Sciences, 5th of Qotb-e Ravandi Blvd, Kashan, 8715973449, Iran, 98 9131638113, 98 31 5510 9999

**Keywords:** continuance intention, expectation–confirmation model, habits, mobile health apps, perceived usefulness, user satisfaction, technology anxiety

## Abstract

**Background:**

Mobile health (mHealth) apps enhance clinical nursing by improving access to resources and patient care. Further benefits include reduced errors, time savings, better communication, cost reduction, and training. Understanding factors driving nurses’ continued mHealth adoption is key to its sustained success.

**Objective:**

This study extends the expectation-confirmation model (ECM) to explore the determinants of Iranian nurses’ continuance intention to use mHealth apps in their daily clinical routines.

**Methods:**

A cross-sectional, descriptive-analytical study was conducted among 315 nurses from hospitals affiliated with Kashan University of Medical Sciences. The Nurses’ Mobile Health Device Acceptance Scale (NMHDA-Scale) was developed by the authors in 2022. The Intention to Continue Using Mobile Health Applications for Nurses questionnaire assesses nurses’ future willingness to use mHealth apps in their practice. This questionnaire was designed based on the ECM and the approach by Waltz et al. Its primary aim is to identify the factors that influence mHealth device acceptance, specifically among clinical nurses, as previous studies have not focused on this group and have shown inconsistent relationships between various factors. Participants completed structured questionnaires measuring perceived usefulness, perceived ease of use, social influence, habits, and technology anxiety. Data were analyzed using structural equation modeling in AMOS (version 26). The model tested relationships among confirmation, perceived usefulness, social influence, technology anxiety, and mHealth continuance behavior.

**Results:**

The analyzed sample (n=315) consisted primarily of female (252/315, 80%) and married (243/315, 77.1%) nurses, with a mean age of 35.67 (SD 1.24) years. The analysis revealed that perceived usefulness was significantly influenced by both confirmation (*P*<.001) and social influence (*P*<.001). Perceived ease of use was negatively impacted by new technology anxiety (*P*<.001), indicating that higher anxiety levels reduced perceived ease of use. Additionally, mHealth continuance behavior was positively associated with habits (*P*=.002), social influence (*P*<.001), and perceived security risks (*P*=.008). Contrary to expectations, perceived usefulness did not directly influence mHealth continuance (*P*=.15), suggesting that other factors, such as habits and social influence, play a more significant role in long-term use.

**Conclusions:**

Sustained mHealth app use by nurses hinges more on social influence and confirmed expectations than perceived usefulness. Although new technology anxiety remains a barrier, habits and social influence are key to long-term adoption. Hospital leaders should prioritize strategies that foster positive social reinforcement, minimize security concerns, and reduce anxiety through training and support when integrating mHealth into nursing workflows. These findings offer critical insights for improving digital health implementation, ultimately enhancing patient care and clinical efficiency.

## Introduction

Clinical nurses are responsible for various care interventions, such as administering prescribed medications, taking vital signs, changing wound dressings, and providing supportive and educational interventions to patients [1]. In this context, smartphones offer a new opportunity to improve the quality of care [2]. The use of smartphones in nursing offers several benefits, such as reducing medication errors, improving time management and communication [3], implementing effective cost-saving strategies [4], and improving the quality of education [5,6]. According to a study conducted in 2021, approximately 80% of nurses use their smartphones in the workplace for personal and professional purposes, using them as useful tools to improve the quality of care [7]. Another study conducted in 2018 reported that approximately 62.4% of nursing students use smartphones, with the most commonly used technological resources being medical dictionaries, anatomical atlases, and nursing care guides [8].

Many hospitals are in the process of developing policies regarding the use of smartphones by health care providers in the clinical setting. However, several studies have suggested that nurses may face challenges with accurately determining the appropriateness of smartphone use in the workplace, which may lead to clinical complications [9]. Many studies have examined the continued use of smartphones by users [10,11]. However, these studies have been criticized for their different methodologies and lack of consistency in prioritizing factors related to smartphone use in care teams [12-14]. For example, the study by Muqtadiroh et al [15] revealed a direct relationship between perceived usefulness (PU) and continuance-using factors. In contrast, the study by Wang et al [16] revealed a positive correlation between perceived ease of use (EU) and continuance-using factors. However, another study conducted in 2016 did not support this hypothesis [17].

In the context of technology adoption, continuous use is often associated with the cognitive process of postadoption behavior. This involves the conscious evaluation of technology tools during their use [18]. A prominent theory often used in research on continuance intention is the expectation-confirmation model (ECM). The theoretical framework proposed by Bhattacherjee [19] has received considerable attention in technology acceptance and continuance research [20,21]. The ECM examines the relationships among confirmation, PU, and user satisfaction (SA) with the continuance-using factor [22]. Due to the lack of similar studies with nurses in Iran, the inconsistent findings of previous research, and the strengths of the ECM in elucidating behavioral intentions, this study was designed and conducted to investigate the factors influencing the intention to continue using mobile health (mHealth) apps among nurses in Kashan, with a special focus on the ECM.

## Methods

### Study Design and Participants

This cross-sectional study was conducted between March 2022 and September 2022 in 6 hospitals affiliated with Kashan University of Medical Sciences. To determine the sample size for structural equation modeling (SEM), based on scientific sources, 5 to 10 samples per item were sufficient to assess the model fit [23]. Given the 33-item scale developed by Mirabootalebi et al [24], 315 clinical nurses were considered as the appropriate sample size. Nurses were selected using systematic sampling. In addition, proportional allocation was used to ensure that the number of nurses selected from each hospital was proportional to the population of nurses in that specific hospital. Sample selection was based on a list provided by the education department of each hospital. In cases where the selected nurses were unavailable or uncooperative, another nurse was randomly replaced (based on the random numbers table).

The inclusion criteria included having at least 6 months of work experience in clinical wards and the use of at least one technology tool in nursing. Exclusion criteria included a failure to complete the data collection scales and withdrawal from further cooperation. After data collection, each scale received was systematically checked for errors, incompleteness, and unanswered items. To ensure the validity of the analysis and an appropriate representation of the data, scales that contained unanswered items or where the response “no comment” was used for all items were excluded from the data analysis. Finally, 368 nurses completed the scales, and after the screening process, data from 315 nurses were considered for data analysis.

### Data Collection Scales

The data collection scales included the Nurses’ Mobile Health Device Acceptance Scale (NMHDA-Scale) and the Intention to Continue Using the Mobile Health Application for Nurses questionnaire.

The NMHDA-Scale was developed by Mirabootalebi et al in 2022 [24]. This questionnaire was designed based on the ECM and the approach by Waltz et al [25]. This questionnaire aims to identify influential factors based on the ECM. Previous studies have not focused specifically on clinical nurses and have shown inconsistent relationships between factors. The questionnaire comprises 10 domains, including perceived security risk (PS), new technology anxiety (AN), social influence (SI), EU, confirmation, mHealth continuance (MHC), maturity (MA), PU, habits (HA), and SA. The reliability of the questionnaire was assessed via Cronbach α and McDonald omega coefficients, which were estimated to be 0.877 and 0.879, respectively [24].

The Intention to Continue Using Mobile Health Applications for Nurses questionnaire is designed to measure nurses’ willingness to continue using mHealth apps for nursing care in the future. It also includes the willingness to use these tools, efforts to improve nursing care with smartphones, and strategies to integrate them into nursing services. The score of this questionnaire is calculated based on the average score of 3 items. Internal consistency was confirmed via the Cronbach α coefficient, combined reliability (CR), and average variance extracted (AVE) to assess discriminant and convergent validity. The Cronbach α coefficient was greater than 0.8. In this study, a 5-point Likert scale was used to rate each item, with each item receiving a score between 1 and 5.

### Research Model

To gain a comprehensive understanding of the postimplementation phase of continued use of information systems (IS continuance), this study developed an ECM research model to explore the key factors that influence the continued use of mHealth in Iran. This model combines Bhattacharjee’s ECM [20,21] and model development in other similar studies [13,26-31]. Considering that the relevant literature focuses mainly on the physician population and the need to adapt the factors to the priorities of Iran and the effective personal, organizational, technical, and social factors, the model was developed based on these criteria. This study acknowledges the perspective by Bhattacharjee [19], which offers a theoretical model of IS continuance that distinguishes between acceptance and continuation behaviors. This model draws parallels between people’s IS continuance use decisions and their frequent purchase decisions using information and communication technology.

**Figure 1.**
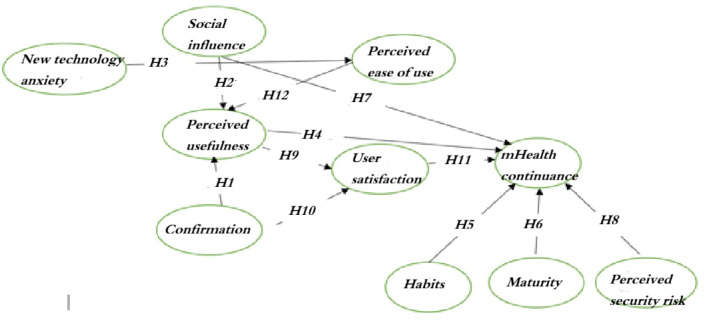
Research model of the research, including the hypotheses (H) linking the constructs.

As seen in Figure 1, confirmation refers to the user’s perception of the fit between the IS expectations and actual performance [13,28]. MA refers to the user’s perception of the quality of the system and reflects the user’s perceived level of satisfaction and the need to improve the system [13]. PU is the extent to which a person believes that his or her job performance will improve with a particular system [13,22]. MHC represents the user’s intention to continue using technology tools [13]. EU is the extent to which a person intends to exert minimal physical and mental effort when using a particular system [29]. PS refers to the ability of a system to protect information from potential threats and to ensure protection from attacks that could compromise data and services [30]. AN refers to the fear of using new technologies [31]. SA is considered an important component of the use and success of an IS [18]. SI refers to the perceived social pressure that influences behavioral decisions [28]. Finally, HA is proposed as an important construct that influences the continuation or discontinuation of technology use [18].

The hypotheses of this study are as follows:

- Hypothesis 1 states that confirmation is related to PU.
- Hypothesis 2 states that SI is related to PU.
- Hypothesis 3 states that AN is related to EU.
- Hypothesis 4 states that PU is related to MHC.
- Hypothesis 5 states that HA is related to MHC.
- Hypothesis 6 states that MA is related to MHC.
- Hypothesis 7 states that SI is related to MHC.
- Hypothesis 8 states that PS is related to MHC.
- Hypothesis 9 states that PU is related to SA.
- Hypothesis 10 states that confirmation is related to SA.
- Hypothesis 11 states that SA is related to MHC.
- Hypothesis 12 states that EU is related to PU.

### Data Analysis

Data were analyzed using SPSS version 26 (IBM Corp) and AMOS version 24 (IBM Corp). Categorical variables are described as frequencies and percentages, whereas quantitative variables are summarized as the means and SDs. Correlations between variables were assessed using the Spearman-Brown correlation coefficient. SEM was used to examine the relationships between the variables influencing the continuous use of mHealth apps. In accordance with the theoretical model of the study, all variables were analyzed using AMOS software to understand the relationships between the variables, and a structural model was developed. In this study, the Pearson correlation coefficient (*r*) and regression (*R*^2^) were used to determine the relationship with each research variable, with the significance level set at <.05. The structural model test was conducted using path coefficients and *R*^2^ values. The *R*^2^ values indicate the percentage of the variability of the internal variables that can be explained by the external variables, thus demonstrating the predictive power of the model. In addition, model fit criteria were examined, including the root mean square error of approximation (RMSEA), comparative fit index (CFI), Tucker-Lewis index (TLI), *χ*^2^ divided by degrees of freedom (*χ*^2^/*df*), incremental fit index (IFI), and goodness-of-fit index (GFI). Goodness of fit was indicated by critical values for *χ*^2^/*df* (<3), CFI and TLI (≥0.9), RMSEA (<0.8), and GFI and IFI (≥0.90) [32,33].

### Ethical Considerations

#### Ethical Statement

The research protocol was submitted to an independent ethics committee to ensure compliance with ethical standards. Any feedback from the committee was addressed prior to the commencement of data collection. All necessary approvals were obtained from the Research Council (number 039, dated 2022) and the Ethics Committee of Kashan University of Medical Sciences (code 40138). The necessary approvals were also obtained from the Vice-Chancellor for Research and Technology of Kashan University of Medical Sciences (IR.KAUMS.NUHEPM.REC.1401.039). This study was conducted in accordance with the principles outlined in the Declaration of Helsinki. We confirm that the research protocol was reviewed and approved by the Institutional Review Board at Kashan University of Medical Sciences.

#### Informed Consent

The clinical nurses provided consent to participate in the study after being informed about the purpose, procedures, potential risks, and benefits of the research; participation; right to withdraw at any time without penalty; and anonymity of the data collected.

#### Confidentiality

All data collected from participants were treated confidentially and anonymized. Personal identifiers were removed to ensure privacy, and data were stored securely, accessible only to the research team.

## Results

### Participant Characteristics

In this study, 400 clinical nurses were included, and data for 315 nurses were analyzed. The mean age of the nurses was 35.67 (SD 1.24) years. Most of the included nurses were female (252/315, 80%) and married (243/315, 77.1%; Table S1 in Multimedia Appendix 1).

### Two-Step Path Analysis Approach

According to the questionnaire scores, MHC was rated as high. SA, PS, affirmation, HA, PU, and MA were all rated as average. In addition, EU, SI, and AN received low scores (Table 1). The Pearson correlation coefficient revealed significant correlations between the variables studied (Table 2). Two-step path analysis was used for the SEM. In the first step, reliability assessment and confirmatory factor analysis were performed. The internal consistency of the questionnaire was assessed using the Cronbach α, CR, and AVE and yielded values of 0.851, 0.892, and 0.63, respectively. These results indicate that the reliability of the questionnaire meets the acceptable thresholds for Cronbach α (>0.7), CR (>0.7), and AVE (>0.5). Therefore, the data were suitable for SEM and correlation analysis (Table 1).

**Table 1.** Means, SDs, reliabilities, and validities of the model variables.

Variables	Mean (SD)	Cronbach α	CR^a^	AVE^b^
Perceived ease of use	15.6 (4.4)	0.888	0.999	0.62
mHealth continuance	11.1 (2.2)	0.763	0.995	0.61
Maturity	9.6 (2.6)	0.908	0.981	0.61
Perceived usefulness	10 (2.2)	0.763	0.817	0.60
Habits	9.6 (2.8)	0.818	0.821	0.60
Social influence	21.5 (4.4)	0.875	0.791	0.52
New technology anxiety	13.3 (2.2)	0.878	0.991	0.63
Perceived security risk	9 (2.76)	0.901	0.922	0.75
Confirmation	9.6 (2.5)	0.861	0.872	0.75
User satisfaction	9.5 (2.5)	0.855	0.722	0.61

a CR: combined reliability.

b AVE: average variance extracted.

**Table 2.** Correlation matrix of the model variables.

Variable	EU^a^	SI^b^	AN^c^	HA^d^	PS^e^	CO^f^	MA^g^	PU^h^	MHC^i^	SA^j^
EU										
*r*	1	0.306	0.699	0.692	0.803	0.737	0.738	0.654	0.323	0.744
*P* value	—^k^	<.001	<.001	<.001	<.001	<.001	<.001	<.001	<.001	<.001
SI										
*r*	0.306	1	0.370	0.374	0.362	0.370	0.347	0.443	0.633	0.334
*P* value	<.001	—	<.001	<.001	<.001	<.001	<.001	<.001	<.001	<.001
AN										
*r*	0.699	0.370	1	0.716	0.715	0.681	0.659	0.602	0.337	0.709
*P* value	<.001	<.001	—	<.001	<.001	<.001	<.001	<.001	<.001	<.001
HA										
*r*	0.692	0.374	0.716	1	0.681	0.660	0.641	0.569	0.360	0.643
*P* value	<.001	<.001	<.001	—	<.001	<.001	<.001	<.001	<.001	<.001
PS										
*r*	0.803	0.362	0.715	0.681	1	0.740	0.697	0.652	0.220	0.705
*P* value	<.001	<.001	<.001	<.001	—	<.001	<.001	<.001	<.001	<.001
CO										
*r*	0.737	0.370	0.681	0.660	0.740	1	0.821	0.741	0.444	0.860
*P* value	<.001	<.001	<.001	<.001	<.001	—	<.001	<.001	<.001	<.001
MA										
*r*	0.738	0.347	0.659	0.641	0.697	0.821	1	0.794	0.430	0.838
*P* value	<.001	<.001	<.001	<.001	<.001	<.001	—	<.001	<.001	<.001
PU										
*r*	0.654	0.443	0.602	0.569	0.652	0.741	0.794	1	0.546	0.781
*P* value	<.001	<.001	<.001	<.001	<.001	<.001	<.001	—	<.001	<.001
MHC										
*r*	0.323	0.633	0.337	0.360	0.220	0.444	0.430	0.546	1	0.408
*P* value	<.001	<.001	<.001	<.001	<.001	<.001	<.001	<.001	—	<.001
SA										
*r*	0.744	0.334	0.709	0.643	0.705	0.860	0.838	0.781	0.408	1
*P* value	<.001	<.001	<.001	<.001	<.001	<.001	<.001	<.001	<.001	—

a EU: perceived ease of use.

b SI: social influence.

c AN: new technology anxiety.

d HA: habits.

e PS: perceived security risk.

f CO: confirmation.

g MA: maturity.

h PU: perceived usefulness.

i MHC: mHealth continuance.

j SA: user satisfaction.

k Not applicable.

A significance level ≤.05 was used to interpret and accept or reject the hypotheses. We concluded that all hypotheses, except hypotheses 4 and 6, were confirmed. Importantly, a positive path coefficient indicates that the change in the 2 variables is in the same and direct direction. Conversely, a negative standard path coefficient indicates that both variables change negatively and vice versa. In other words, when one variable increases, the other variable decreases, or when the independent variable increases, the dependent variable decreases (Table 3).

**Table 3.** Statistical assumptions related to the analysis of the measurement model.

Hypothesis	Path	Path coefficient		Critical value	*P* value
		Standard	Nonstandard		
1	PU^a^ → CO^b^	0.895	0.852	13.9	<.001
2	PU → SI^c^	0.127	0.133	2.99	<.001
3	EU^d^ → AN^e^	0.844	0.952	13.62	<.001
4	MHC^f^ → PU	0.574	−0.543	1.4	.15
5	MHC → HA^g^	0.188	0.124	2.24	.002
6	MHC → MA^h^	−0.364	−0.276	−0.905	.37
7	MHC → SI	0.527	0.521	7.7	<.001
8	PS^i^→MHC	−0.234	−0.184	−2.66	.008
9	PU → SA^j^	15.283	5,666	2.697	.007
10	CO → SA	−15.527	5.738	−2.706	.007
11	SA → MHC	0.574	−0.543	1.4	.15
12	EU → PU	1.004	0.065	15.453	<.001

a PU: perceived usefulness.

b CO: confirmation.

c SI: social influence.

d EU: perceived ease of use.

e AN: new technology anxiety.

f MHC: mHealth continuance.

g HA: habits.

h MA: maturity

i PS: perceived security risk.

j SA: user satisfaction.

### Structural Equation Modeling

**Figure 2.**
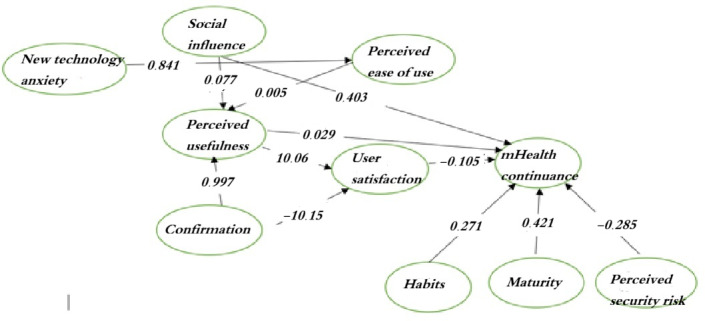
Structural model of the research.

In this study, the initial fit of the model was checked. The fit of the adjusted model was then assessed and validated (Table 4 and Figure 2).

**Table 4.** Fit indices for the measurement model and structural model.

Fit indicators	Recommended value	Measurement model		Structural model	
		Before modification	After modification	Before modification	After modification
*χ*^2^/*df*^a^	1‐3	2.178	1.89	4.771	2.36
CFI^b^	≥0.90	0.925	0.944	0.744	0.910
GFI^c^	≥0.90	2.178	0.854	0.642	0.832
TLI^d^	≥0.90	0.914	0.935	0.724	0.900
IFI^e^	≥0.90	0.926	0.944	0.746	0.911
RMSEA^f^	<0.08	0.061	0.053	0.110	0.066

a *χ*2/*df*: *χ*2 divided by the degrees of freedom.

b CFI: comparative fit index.

c GFI: goodness-of-fit index.

d TLI: Tucker-Lewis index.

e IFI: incremental fit index.

f RMSEA: root mean square error of approximation.

### Measurement Model

The measurement model consists of 33 items distributed across 10 variables: PS, AN, SI, EU, confirmation, MHC, MA, PU, HA, and SA. We used 6 model fit criteria to evaluate the overall fit of the model, including the RMSEA, CFI, TLI, *χ*^2^ divided by degrees of freedom (*χ*^2^/*df*), IFI, and GFI. The statistical results of the model fit are summarized in Table 4.

### Structural Model

The results confirmed 6 hypotheses, including hypotheses 1, 2, 3, 5, 7, and 8. In contrast, hypotheses 4 and 6 did not reach statistical significance and were not supported by this study (Figure 1 and Table 4). EU was influenced by AN (*P*<.001). Furthermore, PU was influenced by confirmation, EU, and SI (*P*<.001). In addition, MHC was influenced by HA (*P*=.002), SI (*P*<.001), and PS (*P*=.008).

## Discussion

### Principal Findings

This study investigated the predictors of Iranian nurses’ intention to continue using ECM-based mHealth apps in professional practice. The results showed that the use of mHealth services by nurses is optimal, with the majority expressing a willingness to use smartphones for health care services. Therefore, mHealth technology is an important tool for improving health care services. The results of this study show a positive influence of the model constructs on smartphone use. Further development of these constructs could improve technology acceptance in nursing [34].

The empirical results of the SEM confirmed the hypothesized model and showed significant relationships between affirmation and PU. Hsiao and Chen [13] defined the PU factor as the user’s understanding of the expected health-related benefits. In addition, the results of the study by Lim et al [35] are consistent with the results of our study.

The results of this study show that nurses recognize the impact of mHealth tools on their professional performance. These tools were shown to facilitate data collection, documentation, and analysis of clinical patient data; increase productivity; improve care delivery; promote communication among nursing team members; and support family-centered care.

The SI factor was the second significant variable associated with the PU of mHealth tools. SI encompasses community preferences and values that can significantly influence user perceptions and attitudes. Importantly, technology acceptance is based not only on individual beliefs but also on SIs such as affiliation and perceived popularity [36].

In this study, nurses acknowledged the significant influence of upstream organizations, including the Ministry of Health, senior hospital managers, academics, nurses, and physicians, on recognizing the benefits of mHealth for the delivery of nursing care. The findings of this study support the assertion by Lwoga and Lwoga [27].

The study also found a significant relationship between AN and the EU factor. Sezgin et al [26] also confirmed a significant relationship between the AN and EU factors. Technology anxiety refers to consumers’ fears when first encountering a new technology, as well as their willingness and ability to adapt to it. This anxiety can lead to reduced technology acceptance and ultimately hinder users’ intention to use the technology [37].

The results of this study can be attributed to the fact that smartphone anxiety can negatively affect the EU factor. Apprehension, fear, and hesitation related to smartphone use can significantly affect EU. Given the busy and responsible nature of the nursing profession, it is understandable that there may be some hesitation due to the fear of making mistakes.

This study did not find a significant relationship between PU and MHC. In other words, the role of PU in influencing nurses’ intention to continue using mHealth was interpreted differently than expected in the conceptual model of this study. Despite the importance of PU for nurses’ intention to continue using mHealth, it did not have the expected impact. Previous studies have shown a significant correlation between these factors [38,39]. These different results may be due to different research areas, user characteristics, and system applications. Since this factor has been confirmed in other studies, it could be due to reasons such as differences in user demographics, contexts, or mHealth system design. Our focus on nurses in health care and the specific system’s usability may explain the differing impact of PU on mHealth use continuation. Furthermore, users’ technological proficiency could also influence their perception of usefulness.

The results of this study suggest a significant correlation between HA and mMHC factors. HA refers to the extent to which a person automatically uses mHealth apps. These findings are consistent with those of a 2019 study by Hsiao and Chen [13]. Individual habits are deeply ingrained behaviors that are triggered by situational cues, such as places, people, and past actions. These automatic behaviors are associated with 2 factors, namely (1) smartphone use and (2) the cues that trigger them [39].

The results of this study indicate that the MA factor pathway did not significantly influence MHC. This study proposes an MA model of mHealth through the ECM to provide a comprehensive understanding of the factors that influence people’s acceptance of mHealth and how their use progresses from early to advanced stages. The results of the study by Hsiao and Chen [13] are inconsistent with those of our study. They suggested that the lack of nurses’ approval may be due to poor quality and dissatisfaction with the technology, leading users to view the system as needing improvement. Studies have shown that poor graphical user interface design and inadequate process design of smartphone systems lead to preventable medical errors [13]. In addition, a lack of understanding of the user interface, user discomfort, and user perceptions of the poor quality of the smartphone system reduce the likelihood of use [13,40]. The development of high-quality user interface designs is critical for mobile learning apps because they can influence user acceptance and usage [41]. In this study, Iranian nurses or users may be in different circumstances than in the study by Hsiao and Chen [13], including differences in experience, technologies used, and even organizational culture, which could be influential because technology at different times and places shows different levels of maturity. In the study by Hsiao and Chen [13], existing apps may have been more developed and therefore more accepted, and this effect of the MA factor may have been noticed more by users.

This study revealed a significant relationship between SI and MHC factors. This finding is consistent with the findings of Chen et al [42]. The study suggests that people are more likely to adopt smartphones for professional use when they observe others doing so, suggesting that influential people play an important role in the perception of new technologies.

The results of this study did not confirm the significant influence of PS on nurses’ MHC. PS refers to the user’s belief in the safety of the technology [43]. A study by Weichbroth and Łysik [44] revealed that smartphone users are increasingly vulnerable to malicious activities, especially when malware apps are installed on their devices, such as smartphones and tablets. These devices store and protect sensitive information, which serve as a deterrent to their use. These results are consistent with the findings of Natarajan et al [45].

This study did not find a significant influence of PU on SA.

The study by Hsiao and Chen [13] demonstrated a significant relationship between PU and SA. However, Denovan and Marsasi [46] showed that PU positively affects satisfaction and negatively affects attitude. EU is key to satisfaction, as it reflects users’ beliefs that a technology or system will be effortless to use. The study by Nuralam et al [47] indicated that PU significantly influences customer satisfaction, though not directly. In addition, Denovan and Marsasi [46] indicated that PU has a significant positive effect on satisfaction and a significant negative effect on attitude.

This study did not find a significant relationship between confirmation and SA. A significant relationship between approval and SA was confirmed in a related study [13]. This difference can be explained by the measurement tools used and the technology’s cultural context.

This study did not find a significant relationship between SA and the continued use of mHealth. In other words, the impact of SA on nurses’ intentions to keep using mHealth was interpreted differently than anticipated in the conceptual model of this study. The study by Hsiao and Chen [13] demonstrated a significant relationship between SA and continued usage. This difference may stem from variations in user type (clinical nurses in this study) and app type. Birkmeyer et al [48] and Nie et al [49] also found a correlation between SA and continued use.

This study confirms the significant influence of confirmation on PU. The results of a study in this regard confirmed the existence of a relationship between factor endorsement and PU, essentially confirming the hypothesis of our study [13].

The results of the study by Daneji and colleagues [50] regarding the effects of PU, endorsement, and satisfaction on the intention to continue using massive open online courses also confirmed the relationship between the 2 factors of endorsement and PU.

This study identified factors influencing nurses’ work-related smartphone use but has limitations. Specifically, incorporating a qualitative component would have broadened the range of identified factors. Qualitative research, known to enhance quantitative findings, could have provided deeper insights and a more comprehensive understanding [51].

Due to the COVID-19 pandemic and nurses’ heavy workloads, this study lacked the time and resources for interviews and other qualitative data collection. Furthermore, the study’s location in Kashan limits the generalizability of findings to other cities or countries. Cultural, structural, and environmental variations, including differences in working conditions, technology access, training, and customs, influence nurses’ smartphone usage and could lead to different outcomes elsewhere.

To overcome these limitations, future research should prioritize national and transnational validation of the NMHDA-Scale. Furthermore, future studies could explore additional influential factors not examined here.

### Strengths and Limitations

This study’s strength is that, although many studies have used mHealth measurement tools across various groups, none have used a tool specifically designed and psychometrically validated for nurses. Our study addresses this gap by using a tool designed and psychometrically validated for Iranian nurses.

This study also had some limitations, such as limiting the review to English-language studies, excluding research in other languages.

This study recommends actions across 5 dimensions: management and structure, process, technical, staff, and skills. In management and structure, recommendations include formulating mobile app deployment policies for the health sector, creating supportive policies for nurses, aligning policies at the Ministry of Health level, defining program values and goals early, promoting culture building, and establishing confidentiality and security policies. Process recommendations focus on clarifying tool usage and monitoring procedures. Technically, the study advises formulating security and confidentiality programs, policies, and standards, as well as evaluation and monitoring standards. Regarding staff and skills, recommendations encompass conducting educational needs and feasibility assessments, reviewing and formulating educational topics, identifying effective tools and solutions, and addressing nurses’ concerns related to anxiety and security. To effectively address these recommendations, a dedicated committee with relevant expertise should be established within the ministry to conduct investigations and make informed decisions.

### Conclusion

Nurses make up a large percentage of hospital staff. Hospitals face potential financial and nonfinancial risks if nurses do not adopt new technologies in their daily work. Since the clinical care of patients depends on the professional skills and experience of nurses, mHealth tools can improve timely access to medical resources for medical staff. As a result, nurses can improve the quality of patient care and streamline their workflow. The results of this study provide valuable insights into the factors that influence nurses’ use of smartphones in their professional roles. In addition, the study sheds light on the challenges and opportunities nurses face when using smartphones. Managers of health care organizations who want to encourage continued smartphone use must fully understand the reasons behind this behavior. Therefore, a better understanding of nurses’ perceptions of smartphone use is needed to develop new implementation strategies. These strategies should reveal users’ attitudes toward smartphones and thus help developers and system administrators improve them and promote their sustainable use.

This study is a useful tool for hospital managers who need to assess the likelihood of success of a new technology. It also helps nurses understand the benefits of use. It focuses on PU, EU, AN, PS, SA, HA, MA, SI, and endorsement. Overall, the results of this study suggest that nurses’ intention to continue using smartphones can only be increased if attention is paid to characteristics related to the maturity of the technology and the user’s perception of its usefulness.

## Supplementary material

10.2196/68048 Multimedia Appendix 1 Demographic characteristics of the sample (n=315).

## Abbreviations

AN: new technology anxiety
AVE: average variance extracted
CFI: comparative fit index
CR: combined reliability
ECM: expectation-confirmation model
EU: perceived ease of use
GFI: goodness-of-fit index
IFI: incremental fit index
IS: information systems
MHC: mHealth continuance
mHealth: mobile health
NMHDA-Scale: Nurses’ Mobile Health Device Acceptance Scale
PS: perceived security risk
PU: perceived usefulness
RMSEA: root mean square error of approximation
SA: user satisfaction
SEM: structural equation modeling
SI: social influence
TLI: Tucker-Lewis index
