# Supplementary material for: Determinants of Nurses’ Continuance Intention to Use Mobile Health Apps in Clinical Nursing Practice: Structural Equation Modeling to Extend the Expectation-Confirmation Model
Source: JMIR Nurs. 2025 Oct 31;8:e68048. doi: 10.2196/68048 (PMC12578357; doi:10.2196/68048)
Supplement: Multimedia Appendix 1 [file nursing-v8-e68048-s001.docx]

**Table S1.** Demographic characteristics of the samples (n=315).

| **Categorical Variables** | | **N (%)** |
| --- | --- | --- |
| **Sex** (M/F) | | 63/252 |
| **Hospital** | Shahid Beheshti | 264 (83.8) |
|  | Naghavi | 20 (6.3) |
|  | Matini | 4 (1.3) |
|  | Samen Al-Hojaj | 27 (8.6) |
| **Marital status** (Married/Unmarried) | | 243.72 |
| **Educational Level** | Undergraduate | 251 (79.7) |
|  | Postgraduate | 64 (20.3) |
| **Ward** | Emergency | 50 (15.9) |
|  | Internal | 91 (28.9) |
|  | General Surgery | 56 (17.8) |
|  | Intensive Care Unit | 41 (13) |
|  | Paediatric | 23 (7.3) |
|  | Operating Room | 11 (3.5) |
|  | Other | 43 (13.6) |
| **Shift Pattern** | Morning | 88 (27.9) |
|  | Evening | 45 (14.3) |
|  | Night | 66 (21) |
|  | Rotating | 116 (36.8) |
| **How satisfied are you with using m-health?** | Dissatisfaction | 46 (14.6) |
|  | Low Satisfaction | 99 (31.5) |
|  | Relative Satisfaction | 127 (40.3) |
|  | Very Satisfaction | 31 (9.8) |
|  | Completely Satisfied | 12 (3/8) |
| **Quantitative Variables** | | **Mean ± SD** |
| **Age** (Year) | | 35.67 ± 1.24 |
| **Work Experience** | | 10.63 ± 6.49 |
| **Time Spent Using Smartphones** | | 3.34 ± 1.88 |
| **How long do you use the internet during the day?** | | 2.41 ± 0.92 |
| **How often do you use m-health in your professional practice?** (Scoring 1-10; 1 being the least used and 10 being the most used) | | 3.40 ± 2.35 |
